# Ascorbic Acid Neuroprotection Against Hippocampal Injury and Gliosis Induced by E621 in Albino Rats Through Modulation of GFAP, Synaptophysin, and Caspase-3

**DOI:** 10.3390/life16081234

**Published:** 2026-07-26

**Authors:** Enas N. Morgan, Ayman M. Mousa, Rasha A. Elmansy, Hanan Seleem, Marwa M. Fawzi, Amany Refaat Mahmoud, Reham Abdulla Aboukhalil, Hagir H. T. Ahmed, Reem A. Younis, Tarek Hamdy Abd-ElHamid, Asmaa Jabeen, Ashwag Alsharidah, Samah M. Abozaid, Abdullah M. Alnuqaydan, Khaled E. A. Soliman, Enas Haridy Ahmed

**Affiliations:** 1 Department of Physiotherapy, College of Applied Medical Sciences, Qassim University, Buraydah 51452, Saudi Arabia; 2 Department of Basic Health Sciences, College of Applied Medical Sciences, Qassim University, Buraydah 51452, Saudi Arabia; ami.alnuqaydan@qu.edu.sa; 3 Department of Histology and Cell Biology, Benha Faculty of Medicine, Benha University, Benha 13518, Egypt; 4 Department of Anatomy and Histology, College of Medicine, Qassim University, Buraydah 51452, Saudi Arabia; r.elmansi@qu.edu.sa (R.A.E.); h.seleem@qu.edu.sa (H.S.); am.mahmoud@qu.edu.sa (A.R.M.); 5 Department of Histology and Cell Biology, Faculty of Medicine, Menoufia University, Menoufia 32511, Egypt; e.haridy@uoh.edu.sa; 6 Department of Basic Medical Sciences, Al Rayan National College of Medicine, Madinah 42541, Saudi Arabia; mm.fawzy@amc.edu.sa; 7 Department of Forensic Medicine and Clinical Toxicology, Faculty of Medicine, Ain Shams University, Cairo 11566, Egypt; 8 Department of Anatomy and Embryology, Faculty of Medicine, Assiut University, Assiut 71515, Egypt; 9 Department of Pathology, College of Medicine, Qassim University, Buraydah 51452, Saudi Arabia; re.aboukhalil@qu.edu.sa (R.A.A.); hag.ahmed@qu.edu.sa (H.H.T.A.); ka.suliman@qu.edu.sa (K.E.A.S.); 10 Department of Internal Medicine, College of Medicine, Qassim University, Buraydah 51452, Saudi Arabia; r.younis@qu.edu.sa; 11 Department of Histology and Cell Biology, Faculty of Medicine, Assiut University, Assiut 71515, Egypt; tabdelhamid@aun.edu.eg; 12 Department of Basic Medical Sciences, Faculty of Medicine, Aqaba Medical Sciences University, Aqaba 77110, Jordan; 13 Department of Physiology, College of Medicine, Qassim University, Buraydah 51452, Saudi Arabia; a.shokatkhan@qu.edu.sa (A.J.); ashriedt@qu.edu.sa (A.A.); 14 Department of Human Anatomy and Embryology, Faculty of Medicine, Minia University, Minia 61519, Egypt; samah.abozaid@mu.edu.eg; 15 Department of Basic Medical Sciences, Faculty of Medicine, Deraya University, New Minia 61768, Egypt; 16 Department of Anatomy, College of Medicine, Hail University, Ha’il 55223, Saudi Arabia

**Keywords:** dentate gyrus, monosodium glutamate, neurotoxicity, ascorbic acid, oxidative stress, antioxidant

## Abstract

Monosodium glutamate (E621) is a common flavor enhancer in highly processed food. Although it makes food more enjoyable, chronic intake may lead to excitotoxicity in brain areas. The current study investigates histological and biochemical neurodegenerative alterations in the rat hippocampus following E621 administration and evaluates the potential neuroprotective properties of ascorbic acid (AA) against E621-induced adverse effects. Forty adult male albino rats were divided into four groups: control group (G1), AA group (G2), E621 group (G3), and AA + E621 group (G4). All animals received a daily intraperitoneal (IP) injection for 30 days. Hippocampal samples were processed and stained with hematoxylin and eosin (H&E), immunostained for GFAP, synaptophysin (a synaptic protein), and caspase-3, and biochemically analyzed for oxidative markers, including malondialdehyde (MDA) and superoxide dismutase (SOD). G3 exhibited significant neurodegenerative changes, characterized by pyknotic granular cells and cytoplasmic vacuolation, with significantly elevated GFAP, synaptophysin, and caspase-3 immunoreactivity in the dentate gyrus (DG). These structural deficits correlated with elevated MDA levels and reduced SOD levels in G3. In contrast, simultaneous administration of AA with E621 resulted in substantial preservation of neuronal morphology, a reduction in caspase-3 immunoreactivity, and a restoration of synaptic vesicle density in G4. E621 induces hippocampal injury by increasing ROS levels and dysregulating GFAP, synaptophysin, and caspase-3. At the same time, AA preserves neuronal integrity and synaptic homeostasis, suggesting its potential role as a protective dietary supplement against brain injury induced by the E621 flavor enhancer.

## 1. Introduction

Highly processed foods with various additives intended to improve palatability and shelf life are increasingly prevalent in our diets. Among these, monosodium glutamate, identified by the European Food Code (E621), is one of the most widely consumed flavor enhancers globally [1].

Worldwide, E621 is an everyday ingredient in the diets of people across various socioeconomic groups, largely because it is present in affordable, popular products such as instant noodles, flavored snacks, and bouillon cubes, as well as soups, chips, frozen dinners, and seasoning blends [2]. E621 is a water-soluble, white, odorless, crystalline powder [3]. It is the sodium salt of the nonessential amino acid glutamic acid, with 78% glutamic acid, 22% sodium, and water [4]. E621 was first identified in 1908, when Japanese chemist Kikunae Ikeda recognized glutamate as the key component of seaweed’s unique flavor and neutralized it with Na to improve its solubility, thereby producing E621 [5]. E621’s unique flavor is mediated by activation of specific taste receptors on lingual taste buds, called umami receptors, which relay taste signals to the brain’s taste nuclei, allowing this taste to be perceived [6]. E621 increases food palatability by enhancing the umami taste [7]. However, there is no recommended daily intake, and it is regarded as a safe food additive for human consumption. In 1987, the Joint FAO/WHO Expert Committee on Food Additives evaluated the safety of glutamate and its salt and assigned it an “acceptable daily intake (ADI) not specified” rating [5]. However, in 2017, the European Food Safety Authority (EFSA) reevaluated the safety of E621 and established an “acceptable daily intake” of 30 mg/kg BW [8].

The brain contains the highest concentration of glutamate, followed by plasma and extracellular fluid [9]. Glutamate is an excitatory neurotransmitter for the central nervous system [10]. New neurotoxicological research demonstrates that persistent consumption of E621 at excessive amounts for prolonged periods causes serious brain disturbances secondary to excitotoxicity. Excess dietary glutamate elevates extracellular glutamate levels in various brain regions, thereby overstimulating ionotropic receptors, including N-methyl-D-aspartate (NMDA) receptors. This overstimulation triggers a massive, abnormal influx of calcium ions into neurons, leading to subsequent neural degeneration [11]. Many reports indicate that E621 acts as an excitotoxin by generating free radicals that promote cell damage and activate pathways, including inflammatory responses and cell death, leading to a variety of detrimental health impacts [12].

Ascorbic acid (AA) is a water-soluble antioxidant found in a wide range of plants, including broccoli, citrus fruits, blackcurrants, and strawberries. Numerous studies have shown that AA exhibits antioxidant and anti-inflammatory effects. AA has been shown to improve a variety of medical disorders, including diabetes, allergies, cardiovascular diseases, neurodegenerative diseases, and infectious diseases [13]. AA has attracted interest as an efficient antioxidant that combats free radicals and preserves mitochondrial membranes. AA can cross the blood–brain barrier (BBB) and neutralize both extracellular and intracellular free radicals because it has a high concentration of electrons that can bind to free radicals, thereby hindering their detrimental effects [14]. Recently, AA supplementation has been shown to exert neuroprotective effects in rats through multiple mechanisms, including antioxidant defense, modulation of neurotransmitters, and modulation of metabolic transport. This may prevent apoptotic pathways, as indicated by Bcl-2, and lessen the neuroinflammatory response, as indicated by Anti-glial fibrillary acidic protein (GFAP) [15].

The hippocampus is a component of the limbic lobe, situated in the medial portion of the temporal lobe, and is involved in various brain functions, including memory, mood, cognitive processes, and spatial orientation. The hippocampal formation is structured into three distinct parts: the hippocampus proprius, the dentate gyrus (DG), and the subicular cortex [16]. The hippocampus proprius is split histologically into four sections (CA1–CA4) based on the density, size, and branching of pyramidal cell axons and dendrites [17]. The DG is a short-folded band of grey matter that extends from the gyrus fasciolaris to the superior surface of the parahippocampal gyrus. The DG contains architectural fascia dentata (FD), which is divided into three layers from superficial to deep: molecular, granular, and polymorphic cell layers [18]. The granule cell layer (GL) contains both granule cells and immature neurons in the subgranular zone (SGZ), one of the stem cell-rich regions of the adult human brain [19]. The hippocampus is highly vulnerable to calcium-mediated brain injury because it has high concentrations of glutamate receptors, particularly in the CA and DG sections. Damage to these sections has been associated with impairment of memory and spatial learning [20]. As the “calcium cascade” develops, it accelerates the formation of reactive oxygen species (ROS) and depletes the brain tissues’ endogenous antioxidant defense mechanisms, including glutathione and superoxide dismutase (SOD). Although the hazards of E621 have been confirmed in numerous studies, its intake continues to rise dramatically due to increased consumption of packaged foods and the widespread use of fast and ready-to-eat products. As these substances become more widely used, concerns about their safety have grown, and research on them is increasing [21]. Thus, studies on commonly consumed protective natural antioxidants, such as AA, are highly required.

Although numerous studies have reported the neurotoxic effects of E621 and the antioxidant effects of AA, few have simultaneously investigated oxidative stress, astrocytic activation, synaptic remodeling, and apoptotic signaling. Furthermore, the relations between these pathological changes in the hippocampus and their contribution to E621-induced neurotoxicity have not been comprehensively investigated. Therefore, the present study was designed to provide an integrated histopathological, immunohistochemical, morphometric, and biochemical assessment of hippocampal injury induced by E621 and to clarify the potential neuroprotective mechanisms of AA through modulation of GFAP, synaptophysin, activated caspase-3, lipid peroxidation, and antioxidant defense.

## 2. Materials and Methods

### 2.1. Experimental Animals and Chemicals

Forty adult male Wistar albino rats (175–210 g) were utilized. To reduce sampling bias, rats were randomly assigned to four experimental groups by a computer-generated randomization process. Rats were assigned identification numbers from 1 to 40, and a computer-generated random sequence was generated using Microsoft Excel’s random number generator. Animals were subsequently assigned to four experimental groups (n = 10 per group) in the sequence determined. The sample size was determined using prior comparable experimental studies and ethical criteria to minimize animal use. Animals were housed in the animal facility under standard conditions (12 h light/dark cycle) with free access to a standard pellet diet and water. AA tablets (500 mg) were purchased from Nature’s Bounty (Bohemia, NY, USA), and E621 was purchased from Badia Spices (Doral, FL, USA).

### 2.2. Experimental Design and Ethical Approval

The rats were randomly divided into four equal groups (*n* = 10) and treated via intraperitoneal (IP) injection for 30 consecutive days. The control group (G1) was injected with 1 mL of distilled water (DW), which was used as a vehicle because it can dissolve either AA or E621, and the AA group (G2) was injected with AA (500 mg/kg/day) dissolved in 1 mL of DW. E621 group (G3) was injected with E621 (4 mg/kg) dissolved in 1 mL of DW [22], and the combined AA + E621 group (G4) received the aforementioned doses of AA and E621 in 1 mL of DW. All procedures in the current study were approved by the Institutional Animal Care and Use Committee (IACUC) of Deraya University (Number DCSR-02026-100), in accordance with the ARRIVE Essential 10 and the National Institutes of Health (NIH Publication) guidelines for the Care and Use of Laboratory Animals.

E621 and AA were administered IP to provide complete systemic bioavailability by bypassing gastrointestinal absorption. This route allows rapid attainment of high plasma levels necessary to induce hippocampal neurotoxicity. AA is administered via IP to provide immediate, standardized antioxidant protection, bypassing first-pass intestinal metabolism.

### 2.3. Biochemical Analysis

The lipid peroxidation marker, malondialdehyde (MDA), was measured in brain tissues by the thiobarbituric acid assay for colorimetric detection, with 1,1,3,3-tetramethoxypropane as the reference standard [23], and the results were expressed as nmol/g tissue. SOD levels were also measured using a commercial kit (Biodiagnostics, Cairo, Egypt; Cat. No. SD 25 21) according to the manufacturer’s instructions, and results were expressed as U/g protein.

### 2.4. Histological and Immunohistochemical Procedures

Following the treatment period, brain specimens from each rat were fixed in 10% neutral buffered formalin. Paraffin sections (4 µm) were prepared and stained with Hematoxylin and Eosin (H&E) to assess the general morphology of the hippocampus, GFAP immunohistochemistry (IHC) to evaluate astrocyte activation (neuroinflammation), anti-synaptophysin IHC to determine synaptic density and integrity, and anti-caspase-3 IHC to detect caspase-mediated apoptosis.

Paraffin-embedded brain sections were mounted on positively charged slides, deparaffinized, rehydrated, and heated in citrate buffer for antigen retrieval. Endogenous peroxidase activity and nonspecific binding were blocked using 3% hydrogen peroxide. Sections were incubated overnight at 4 °C with primary antibodies against GFAP 1:100 (MS-280-B0; Lab Vision Corporation; Fremont, CA, USA), synaptophysin (A6344; Abclonal Technology; 1:100), and caspase-3 1:100 (PA1-29157; Thermo Fisher Scientific; Waltham, MA, USA). Then, all sections were washed with PBS, incubated with the appropriate secondary antibody, visualized with DAB, and counterstained with Mayer’s hematoxylin. The appropriate positive controls were used, and negative controls were processed by omitting the primary antibody.

Biochemical, histopathological, immunohistochemical evaluation, and morphometric measurements were performed by independent investigators blinded to treatment conditions.

### 2.5. Morphometric Analysis

Ten non-overlapping fields per section were subjected to quantitative morphometric analysis with ImageJ1.54j (NIH, Bethesda, MD, USA) at 400× magnification on an Olympus CX41 light microscope (*Olympus Corporation, Tokyo, Japan*). The percentage of degenerated neurons relative to the total number of neurons in the DG was determined from H&E-stained sections. To calculate the mean area (%) of GFAP, synaptophysin, and caspase-3 immunoreactivity in immunohistochemical sections, color images were converted to 8-bit grayscale, background noise was subtracted, and an automated threshold (Otsu’s method) was applied consistently across all groups to segment positive pixels. The mean area % of immunoreactivity was then determined using the ‘Analyze Particles’ function.

### 2.6. Statistical Analysis

The percentage of degenerated neurons, the mean area percentage (%) of GFAP, synaptophysin, and caspase-3 immunoreactivity, and the levels of MDA and SOD were analyzed using GraphPad Prism 9. Statistical differences between groups were assessed using a one-way ANOVA, followed by the Tukey–Kramer post hoc test for multiple comparisons. A *p*-value < 0.05 was considered statistically significant.

### 2.7. AI-Assisted Language Improvement

The authors used ChatGPT (OpenAI, USA; version 5.5, 2025) as an AI-assisted writing tool to improve the manuscript’s English clarity and refinement. The authors carefully reviewed the content and take full responsibility for the manuscript’s final content and integrity, including the accuracy of the data, interpretations, and conclusions.

## 3. Results

### 3.1. Histopathological Findings of H&E-Stained Sections of the Hippocampus

**Figure 1:**
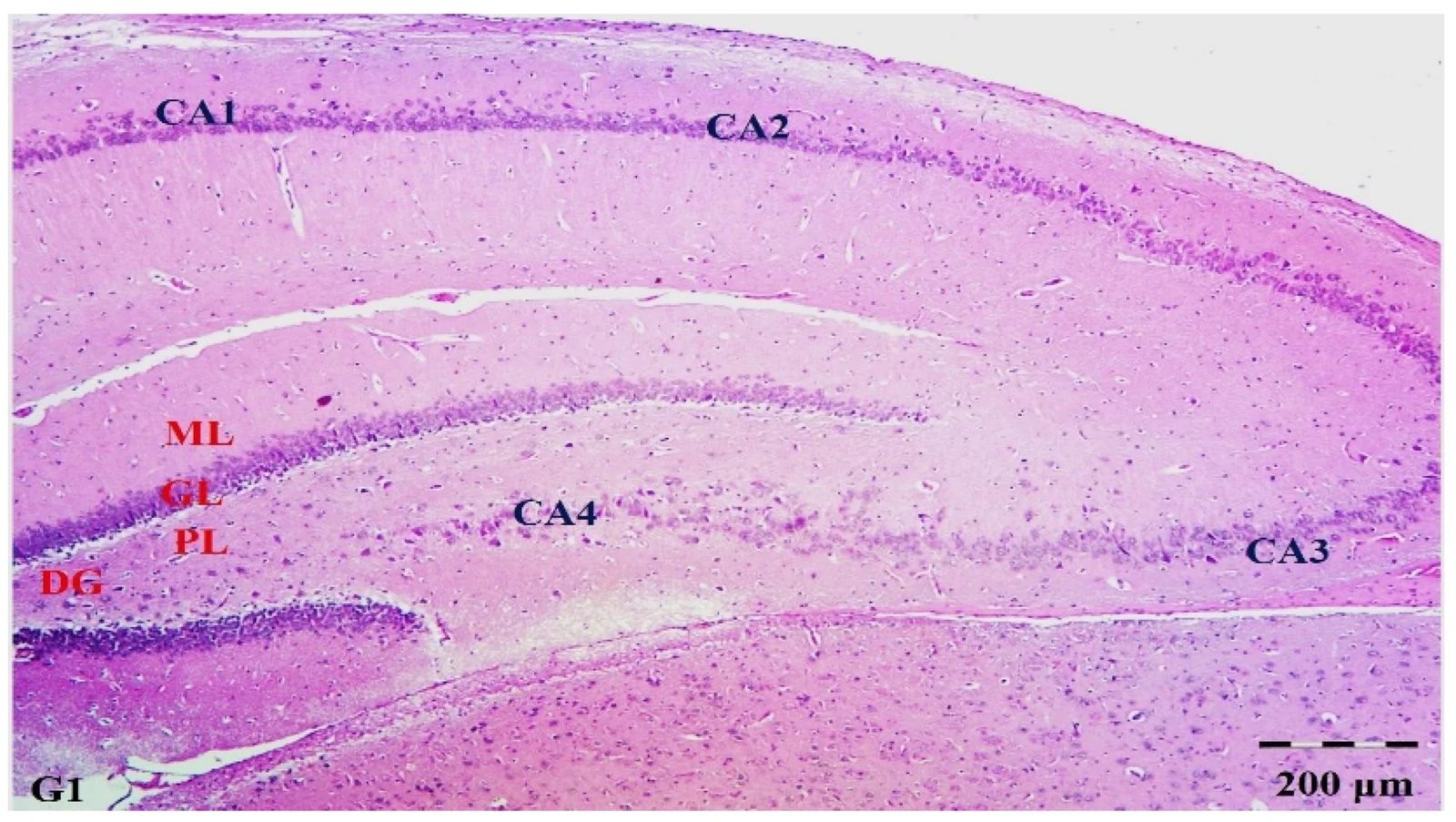
A lower-magnification view of a sagittal section through the hippocampus proper and dentate gyrus (DG) of the control rat group (G1). The normal histological architecture of the hippocampus proper comprises four regions of cornu ammonis (CA1–CA4), and the DG, which appears as a dark V-shaped structure enclosing the CA4 area in G1. The DG has a molecular layer (ML), a granular cell layer (GL), and a polymorphic layer (PL). (H&E 40×).

A sample of H&E-stained sections from the normal hippocampus proper and the DG is shown in Figure 1. The hippocampus exhibited normal histological architecture, comprising four regions of the Cornu Ammonis (CA1–CA4) and the DG (a dark V-shaped structure that encloses CA4).

**Figure 2:**
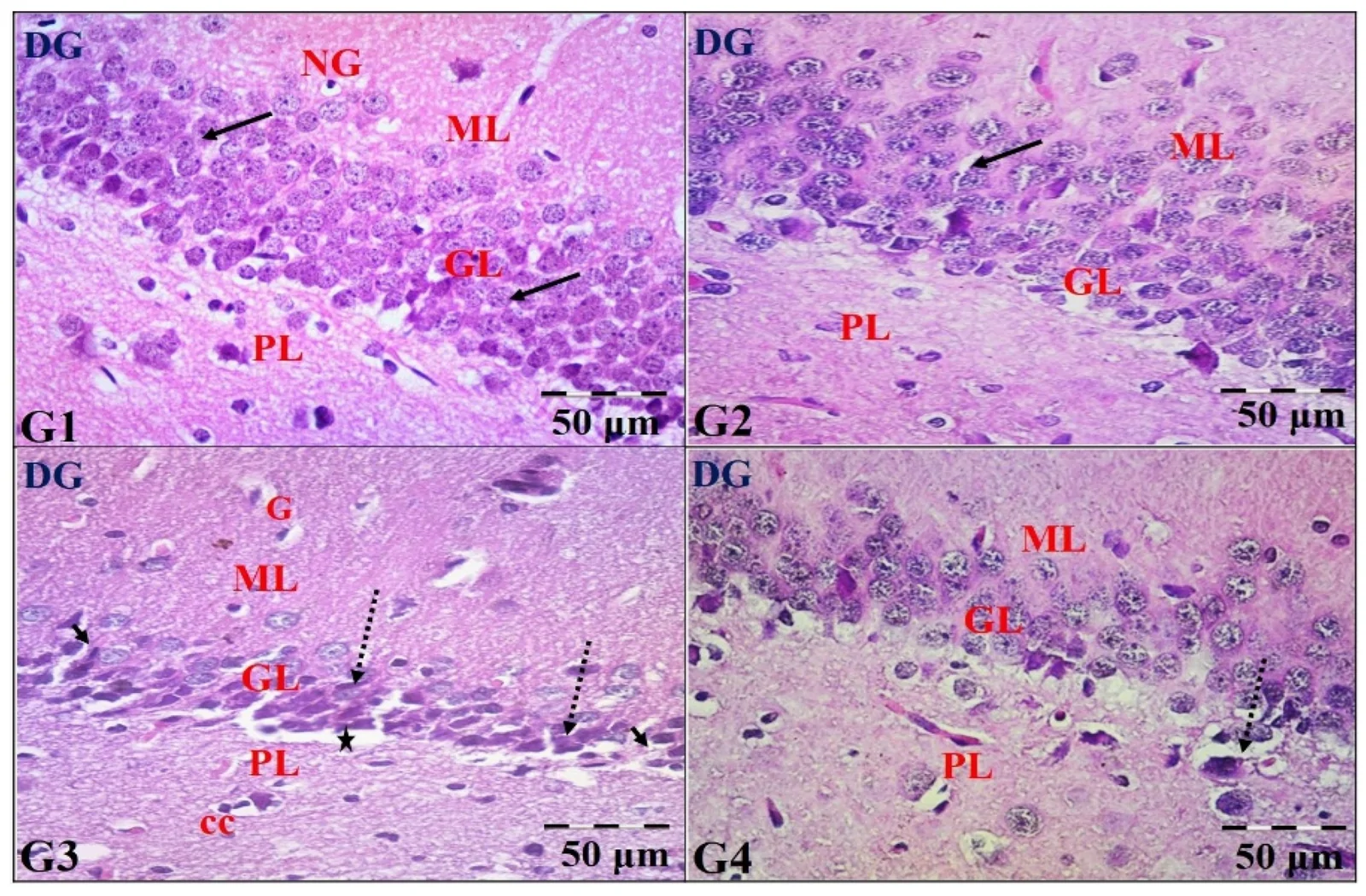
A higher-magnification view of the DG from rats in all studied groups. The DG of G1 (control group) and G2 (AA group) consists of the ML, the GL, and the PL. The GL shows densely packed small granular cells with vesicular nuclei (arrow). Also, sparse neuroglial cells (NG) are scattered within the neuropil, with small, darkly stained nuclei and minimal interstitial tissue in G1 and G2. In contrast, the GL of DG in G3 (E621 group) shows significant histopathological changes, with numerous dark, shrunken granular cells with pyknotic nuclei (dotted arrows), pericellular halos (arrowheads), cytoplasmic vacuolation, and regions of cellular loss (star). As well, the PL in G3 shows extensive neuronal degeneration, with disordered, widely spaced, shrinking cells, pyknotic nuclei, and perineuronal halos, along with congested capillaries (cc), while some neurons appear intact. Also, co-administration of AA with E621 in G4 (AA and E621 group) significantly reduced the histopathological alterations induced by E621. Some granular cells maintain their usual shape, with vesicular nuclei and basophilic cytoplasm, giving them an almost normal appearance, whereas other granular cells in the DG show degeneration and pericellular halos (dotted arrows). (H&E 400×).

Figure 2 shows a higher-magnification view of the DG in G1 and G2, comprising the molecular layer (ML), granular cell layer (GL), and polymorphic layer (PL). The GL showed densely packed small granular cells with vesicular nuclei and minimal interstitial tissues. In contrast, Figure 2 reveals significant histopathological changes in G3, indicating neurotoxic effects of E621. Figure 2 exhibits numerous dark, shrunken granular cells with pyknotic nuclei, pericellular halos, cytoplasmic vacuolation, and regions of cellular loss in the DG. Also, the PL had extensive neuronal degeneration, with disordered, widely spaced, shrinking cells, pyknotic nuclei, and perineuronal halos, along with congested capillaries, while few neurons appeared intact. In contrast, Figure 2 reveals that co-administration of AA with E621 significantly reduced the histopathological alterations induced by E621 in G4, indicating its neuroprotective effects. Most granular cells maintain their typical vesicular nuclei and basophilic cytoplasm, giving them a normal appearance, whereas a few granular cells in the DG of G4 show degeneration and pericellular halos.

### 3.2. Immunohistochemical Staining of GFAP in the DG

**Figure 3:**
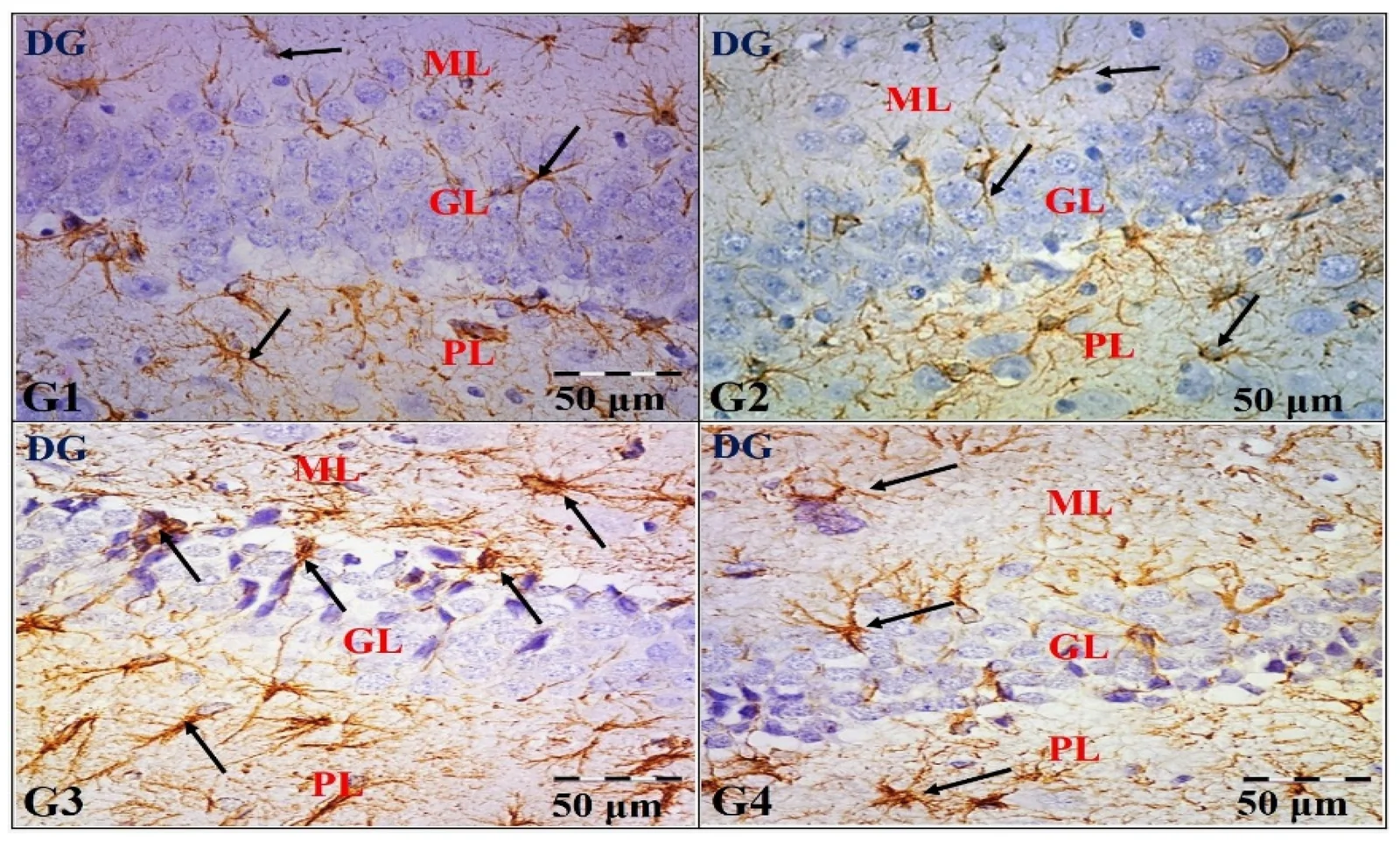
Photomicrographs from DG sections stained with GFAP in all studied groups. A few small astrocytes with thin processes (arrows) and mild astrocytic positivity are dispersed among the cells of ML, GL, and PL in the DG of G1 and G2 sections. In contrast, numerous large astrocytes with thick, multiple, ramified processes (arrows) appear among the cell bodies of the ML, GL, and PL in G3. Also, small astrocytes with few short, thin processes (arrows) are present in the ML, GL, and PL of G4. (GFAP immunostaining 400×).

Figure 3 reveals GFAP immunostaining in the DG region of all groups. Sections from the DG of G1 and G2 revealed thin processes in the GL and PL, along with mild astrocytic positivity. In contrast, G3 sections displayed numerous hypertrophic astrocytes with thick, ramified processes and high GFAP expression, indicating obvious astrocytic activity. Also, G4 sections exhibited small astrocytes with short, thin processes, resulting in a significant reduction in GFAP immunoreactivity after co-administration of AA with E621.

### 3.3. Immunohistochemical Staining of Synaptophysin in the DG

**Figure 4:**
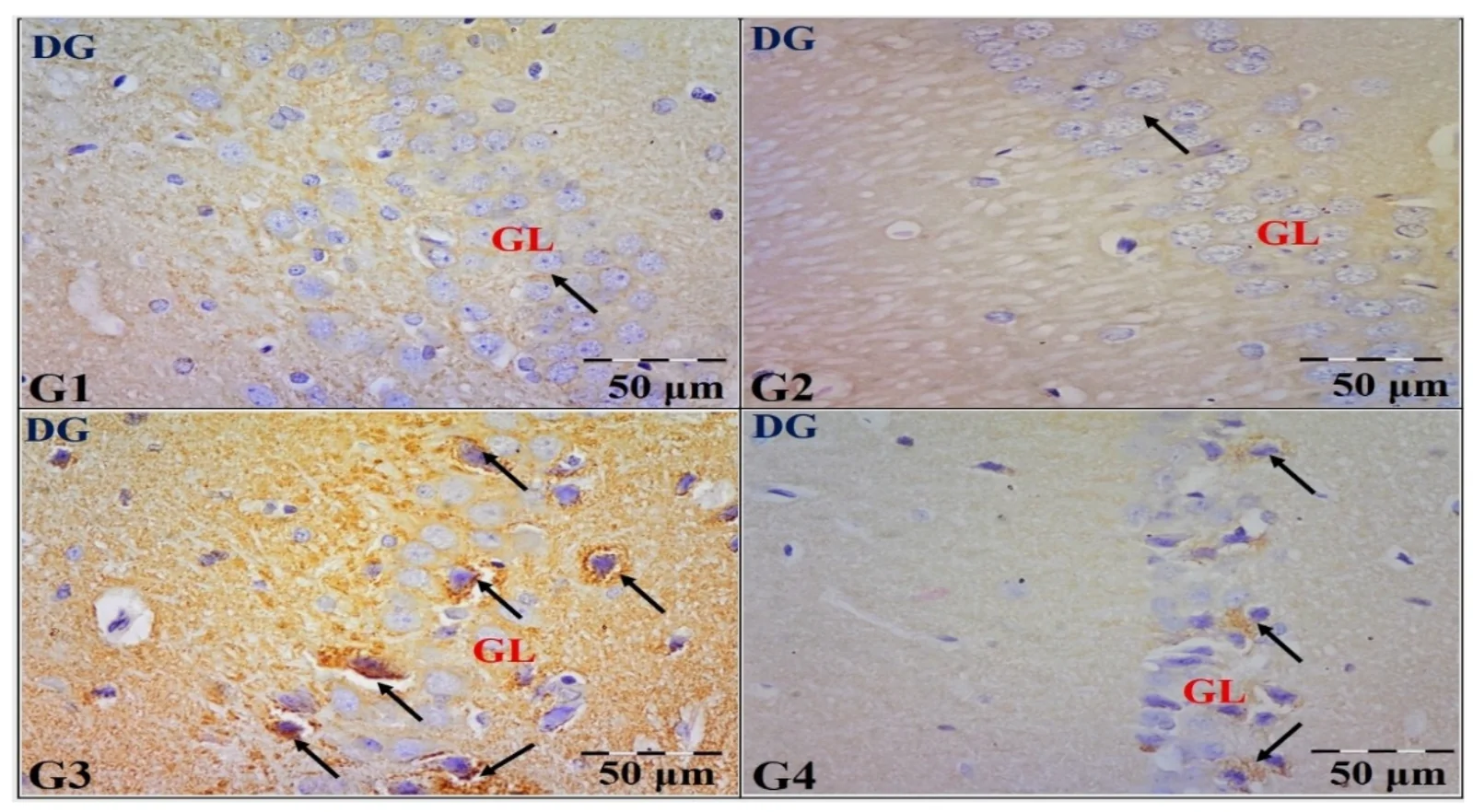
Representative photomicrographs of synaptophysin-immunostained sections in the DG of all studied groups. Synaptophysin appears minimal in the GL (arrow) of G1 and G2; however, it is significantly elevated, with robust, prominent cytoplasmic expression (arrows) in the GL of G3. In contrast, synaptophysin is significantly reduced in the GL (arrows) of G4 compared with G3. (synaptophysin immunostaining 400×).

Figure 4 reveals synaptophysin immunostaining in the DG region of all studied groups. Synaptophysin immunostaining was minimal in the GL of G1 and G2; however, it was significantly elevated in the GL of G3, with robust cytoplasmic expression, indicating that E621 has neurotoxic effects. In contrast, synaptophysin was significantly reduced in the GL of G4 compared with G3, suggesting that coadministration of AA with E621 protects against its neurotoxic effects.

### 3.4. Immunohistochemical Staining of Activated Caspase-3 in the DG

**Figure 5:**
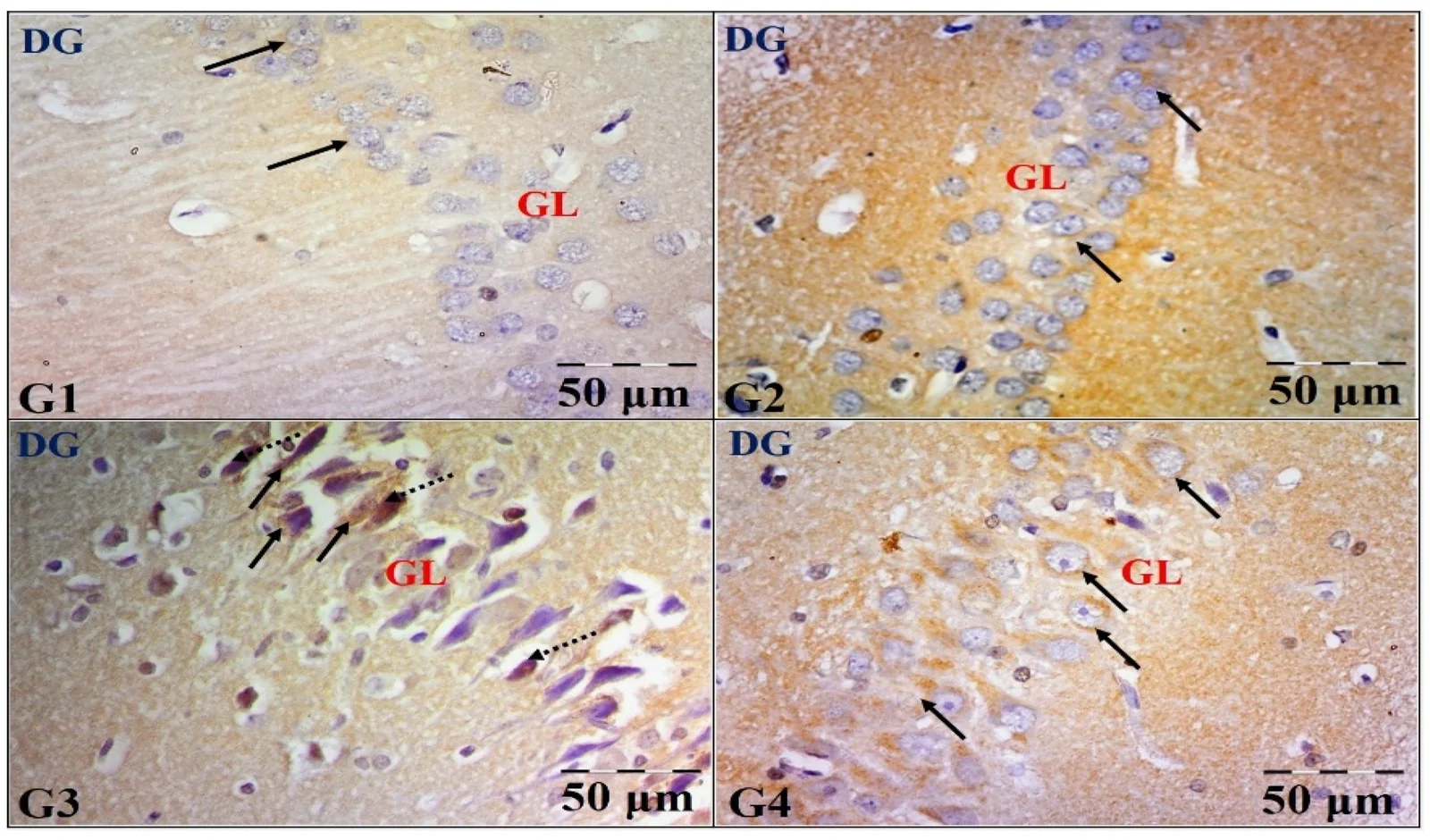
Representative photomicrographs of activated caspase-3 immunostained sections in the DG region of all studied groups. Activated caspase-3 appears faint in the cytoplasm (arrows) of the GL in G1 and G2; however, it is significantly elevated, with strong cytoplasmic (arrows) and nuclear (dotted arrows) expression in the GL of G3. In contrast, activated caspase-3 is significantly reduced with moderate cytoplasmic expression (arrows) in the GL of G4. (activated caspase-3 immunostaining 400×).

Figure 5 shows caspase-3 activation in the DG region across all studied groups. Activated caspase-3 immunostaining was minimal in the GL of G1 and G2; however, it was significantly elevated with cytoplasmic expression in the GL of G3, indicating the neurotoxic effects of E621 in G3 compared with G1 and G2. In contrast, activated caspase-3 was significantly reduced in the GL of G4 compared with G3, indicating that AA administration protects against E621-induced neurotoxicity.

### 3.5. Results of Morphometric Analysis

Table 1 presents the morphometric analysis of the percentage of degenerated neurons and the immunoreactivity of GFAP, synaptophysin, and activated caspase-3 in the DG region of the hippocampus across all studied groups. Histopathological scoring in G3 showed a significant increase in the percentage of degenerated neurons compared to G1 and G2, whereas G4 showed a significant decrease compared to G3, indicating the neuroprotective role of AA administration in G4. At the same time, the surface area for GFAP, synaptophysin, and activated caspase-3 immunoreactivity showed a significant increase in G3 compared with G1 and G2, and a significant decrease in G4 compared with G3, indicating a neuroprotective role for AA against the neurotoxic effects of E621 in G3.

### 3.6. Impact of E621 on Levels of MDA and SOD of Brain Tissue

MDA concentrations in the brain tissues of G3 were substantially higher than in G1, whereas SOD concentrations were significantly lower in G3 than in G1. However, MDA and SOD levels were considerably higher in G4 than in G3 (Table 2).

**Figure 6:**
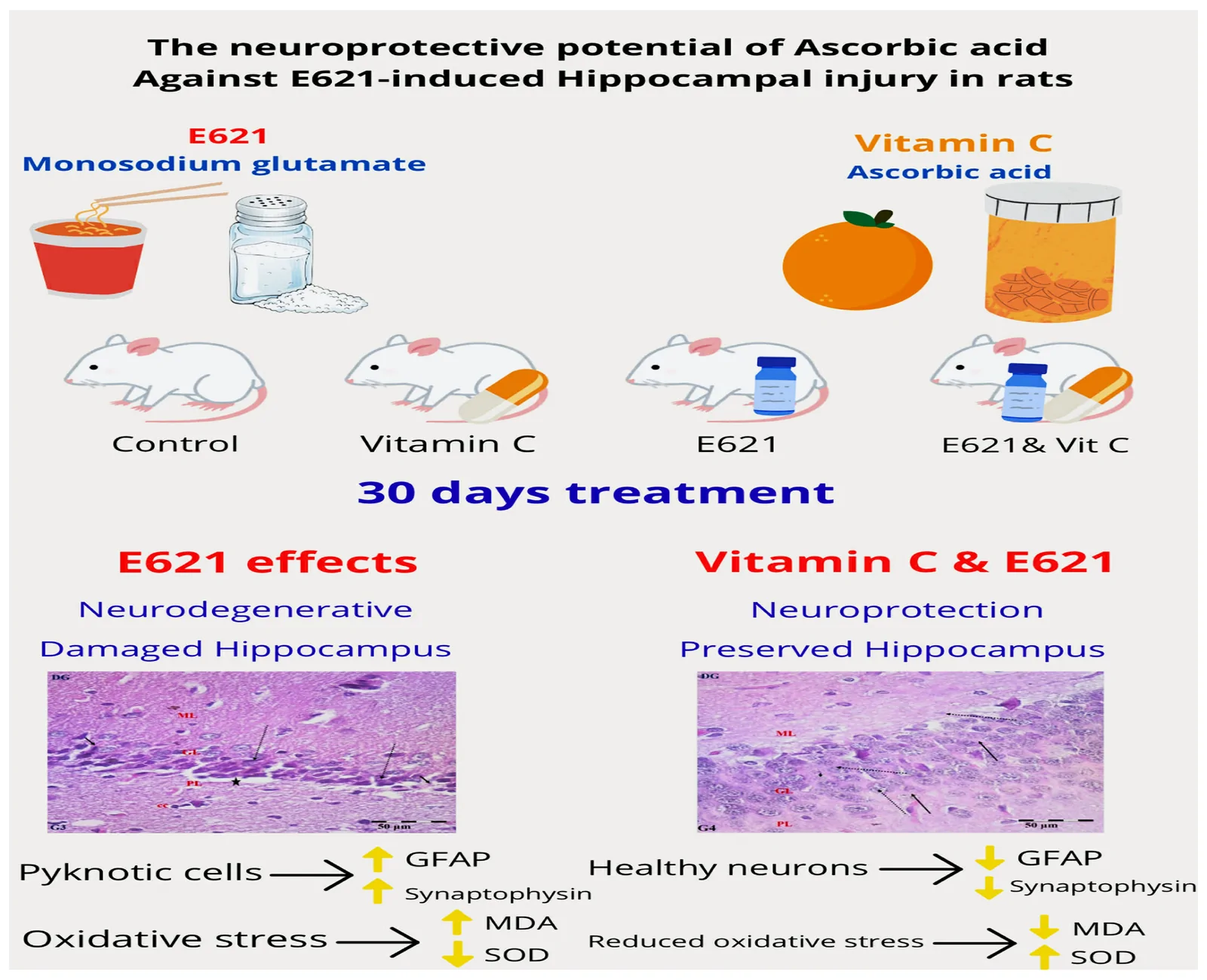
Summarizes the neuroprotective potential of AA against E621-induced hippocampal injury.

Summary of the neuroprotective potential of AA against E621-induced hippocampal injury in Figure 6.

## 4. Discussion

The hippocampus is particularly vulnerable to excitotoxic injury due to its dense glutamatergic circuitry and high metabolic demand, rendering it highly sensitive to E621 consumption [24]. The current study demonstrates that E621 administration induces pronounced histological and biochemical alterations in the hippocampal tissues, while AA co-treatment markedly attenuates these deleterious effects. At the same time, E621 administration was associated with increased levels of the lipid peroxidation product MDA and decreased SOD activity in brain tissues; in contrast, co-administration of AA resulted in a significant decrease in MDA and an increase in SOD activity.

Experimental models of E621-induced neurotoxicity showed considerable variations in dose, route, and duration [11,12,22,24]. Therefore, dose selection depends on the specific objectives and design of each study. The present study employed a chronic low-dose protocol to evaluate the cumulative neurotoxic effects of prolonged MSG exposure and the potential neuroprotective effects of AA.

According to [25], oxidative stress is triggered by an imbalance of antioxidants and oxidants. Excessive production of free radicals, including ROS and nitrogen-derived species, may adversely affect functionally essential molecules, such as genetic material (DNA), lipids, and proteins. Damage to these molecules causes diseases secondary to increased free radical levels in various organs, including the brain, thereby oxidizing proteins, lipids, and nucleic acids [26]. The brain’s high energy requirements generate free radicals through mitochondrial metabolism, which can damage fatty acids and trigger lipid peroxidation.

AA primarily maintains the structural integrity of neural membranes by minimizing lipid peroxidation, as evidenced by lower MDA levels [14]. These neuroprotective findings of AA against neurological damage in rats were also noticed by [27]. Some studies explain the neuroprotective effect of AA by scavenging oxygen-free radicals; additionally, AA has been shown to exert an antiapoptotic effect by increasing catalase activity and reducing lipid levels. Because it can cross the blood–brain barrier and support amino acid synthesis, AA may have a neuroprotective effect [14].

Histological preservation of the DG regions in G1 and G2 confirms maintenance of normal hippocampal cytoarchitecture. In contrast, animals of G3 exhibited characteristic neurodegenerative changes, including neuronal shrinkage, pyknotic nuclei, pericellular halos, cytoplasmic vacuolation, and neuronal loss. These features are consistent with glutamate-mediated excitotoxicity and oxidative stress, which have been repeatedly implicated in E621-induced hippocampal injury [24]. The marked vulnerability of DG granular neurons observed in the present study aligns with their high expression of glutamate receptors and susceptibility to calcium overload and mitochondrial dysfunction. Reactive astrogliosis was evident in the E621 group, as demonstrated by increased GFAP immunoreactivity. Astrocyte hypertrophy is a hallmark of neuroinflammation secondary to neuronal injury. While astrocytic activation initially serves a protective function, sustained gliosis may exacerbate synaptic dysfunction and neuronal loss [28,29]. The reduction in GFAP expression in G4 following AA co-treatment indicates attenuation of the inflammatory response, likely through antioxidant-mediated suppression of oxidative stress.

Synaptophysin immunoreactivity was weak in G1 and G2, significantly elevated in G3, and modestly expressed in G4 following AA co-treatment. Under physiological conditions, low basal synaptophysin expression reflects stable synaptic turnover. The pronounced upregulation observed in G3 likely represents pathological synaptic dysregulation rather than enhanced synaptic function. Recent evidence indicates that excitotoxic and oxidative insults induce abnormal accumulation of presynaptic proteins as a compensatory or maladaptive response to neuronal injury, synaptic loss, or altered vesicle cycling [30]. The reduction in synaptophysin expression to a modest level following AA co-treatment suggests partial restoration of synaptic balance and attenuation of excitotoxic synaptic stress in G3. Also, ref. [31] stated that excitotoxic stimulation activates distinct pathogenic and protective expression signatures in the hippocampus.

The increased synaptophysin immunoreactivity in the present work should not be mistaken for enhanced synaptic function. As ref. [30] reported, excessive glutamate stimulation drives intracellular calcium overload, oxidative stress, and mitochondrial dysfunction, thereby accelerating apoptotic pathways and neurodegeneration. Therefore, when the present work was observed alongside astrogliosis and caspase-3 overexpression, the elevated synaptophysin levels in G3 likely reflect a compensatory or aberrant structural remodeling secondary to severe synaptic disruption. On the other hand, ref. [32] reported that an E621 IP injection did not alter synaptophysin levels. The discrepancy in the impact of elevated glutamate on synaptophysin sensitivity across experiments may be explained by variations in the duration, dose, and route of E621 administration.

Recently, investigating the apoptotic signaling implicated it strongly in E621-induced neurotoxicity, and the marked upregulation of activated caspase-3 in neurons corresponds with the observed nuclear pyknosis and neuronal shrinkage, confirming the activation of programmed cell death pathways. Similar findings have been reported in E621 neurotoxicity models, in which excessive excitatory signaling secondary to glutamate excess triggers mitochondrial dysfunction and caspase-dependent apoptosis [33,34]. Based on a recent investigation, glutamate-induced neuronal cell death is mediated by two distinct pathways: necrotic and apoptotic, with excitotoxic and oxidative damage [32]. The significant reduction in caspase-3 immunoreactivity in G4 highlights the anti-apoptotic role of AA.

Collectively, the findings of the current work indicate that E621 induces hippocampal neurotoxicity through a combination of oxidative stress, astrocytic activation, synaptic dysregulation, and apoptosis. Also, AA exerts a protective effect by mitigating oxidative stress, modulating apoptotic pathways, and stabilizing synaptic protein expression, highlighting its potential therapeutic value against glutamate-mediated neurotoxicity.

The current findings demonstrate that E621 induces severe hippocampal injury via a multi-faceted toxic cascade. Similarly, ref. [11] observed that neuronal pyknosis is a direct consequence of calcium-induced protease activation, which dismantles the neuronal cytoskeleton. Also, ref. [2] reported that GFAP upregulation indicates that E621 triggers a pro-inflammatory state in the brain glial network, thereby exacerbating tissue damage.

AA also exerts its neuroprotective effects in the rat brain through a multifaceted approach involving antioxidant defense, metabolic transport, and neurotransmitter regulation. Primarily, AA acts as a potent antioxidant, scavenging ROS and reducing lipid peroxidation, as evidenced by decreased MDA levels, thereby preserving neuronal membrane integrity [35].

Although AA is hydrophilic, it crosses the blood–brain barrier (BBB) primarily in its oxidized form, dehydroascorbic acid (DHA), utilizing glucose transporters such as GLUT1; once within the brain parenchyma, it is recycled back into its active antioxidant form, AA, to maintain high local concentrations [36]. Beyond its role in oxidative protection, AA functions as a neuromodulator by regulating the release and uptake of glutamate and dopamine. Inhibiting excessive glutamate signaling prevents excitotoxicity, a common pathway for neuronal death in trauma and disease [37]. Furthermore, AA supplementation has been linked to enhanced synaptic plasticity, specifically by facilitating Long-Term Potentiation (LTP) in the hippocampus, which supports the preservation of cognitive function and memory [38].

## 5. Conclusions and Limitations of the Study

The E621-induced hippocampal injury is not the result of a single pathological process; instead, it is most probably due to a coordinated cascade involving oxidative stress, astrocytic activation, synaptic remodeling, and apoptosis. The increased levels of MDA together with the depletion of SOD indicate high oxidative stress, which may initiate neuronal membrane damage and mitochondrial dysfunction. These oxidative changes likely promote astrocyte activation, as reflected by increased GFAP immunoreactivity, and simultaneously disrupt synaptic homeostasis, leading to alterations in synaptophysin expression. Prolonged oxidative stress and excitotoxic signaling ultimately activate caspase-3-dependent apoptotic pathways, leading to neuronal degeneration within the dentate gyrus. The attenuation of these pathological changes with AA administration supports the concept that its neuroprotective effect extends beyond simple antioxidant activity to include the preservation of neuronal integrity, the maintenance of synaptic architecture, and the suppression of apoptosis.

These results suggested that dietary antioxidants could serve as a potential strategy to mitigate food additive-associated neurotoxicity in this experimental model. In light of our findings, a translational toxicology approach linking experimental findings to dietary oral exposure patterns, including the effects of commonly consumed food additives, is needed, and further work is required to investigate the clinical significance of antioxidant supplementation for neuroprotection.

Further molecular studies using techniques such as Western blotting or quantitative PCR, along with behavioral assessments, are recommended to strengthen the mechanistic interpretation of the findings’ functional outcomes. In addition, further investigations are required about the potential gender-related differences in susceptibility to E621-induced neurotoxicity and responsiveness to AA.

## Figures and Tables

**Table 1:** M ± SEM of the percentage of degenerated neurons and the immunoreactivity of GFAP, synaptophysin, and activated caspase-3 in the DG region of the hippocampus across all groups.

Groups (*n* = 10)	M ± SEM of Degenerated Neurons%	*p*-Value	M ± SEM of GFAP IR	*p*-Value	M ± SEM of Synaptophysin IR	*p*-Value	M ± SEM of Activated Caspase-3 IR	*p*-Value
G1	3.80 ± 0.58		17.09 ± 0.65		2.27 ± 0.31		11.13 ± 0.72	
G2	4.79 ± 0.37	0.847 #	20.67 ± 0.39	0.06 #	2.84 ± 0.25	0.463 #	10.69 ± 0.56	0.963 #
G3	29.00 ± 1.01	0.001 # 0.004 &	36.44 ± 1.50	0.001 # 0.003 &	14.35 ± 0.21	0.001 # 0.008 &	20.25 ± 0.66	0.001 # 0.002 &
G4	6.75 ± 1.24	0.053 # 0.001 *	26.02 ± 0.77	0.061 # 0.001 *	8.31 ± 0.32	0.055 # 0.001 *	15.37 ± 0.67	0.064 # 0.001 *

The mean ± standard error of means (M ± SEM), immunoreactivity (IR), # versus G1, * versus G2, & versus G3, *p* < 0.05 is significant.

**Table 2:** Shows the mean ± standard error of the mean (M ± SEM) of MDA & SOD levels in all groups.

Groups (*n* = 10)	M ± SEM of MDA Levels	*p*-Value	M ± SEM of SOD Levels	*p*-Value
G1	25 ± 1.00		67.6 ± 1.778	
G2	21.8 ± 1.53	0.1181 #	61.2 ± 2.154	0.0511 #
G3	52.8 ± 1.428	<0.0001 # <0.0001 *	27.8 ± 1.068	<0.0001 # <0.0001 *
G4	28.2 ± 0.5831	0.0245 # 0.0045 * <0.0001 &	43.2 ± 1.281	<0.0001 # <0.0001 * <0.0001 &

The mean ± standard error of means (M ± SEM), # versus G1, * versus G2, & versus G3, *p* < 0.05 is significant.

## Data Availability

The data that support the findings of this study are available from the corresponding author upon reasonable request.
